# Building up the inhibitory synapse

**DOI:** 10.3389/fncel.2012.00062

**Published:** 2013-01-09

**Authors:** Enrico Cherubini, Paola Zacchi

**Affiliations:** Department of Neuroscience, Scuola Internazionale Superiore di Studi AvanzatiTrieste, Italy

“Building up the inhibitory synapse” is a complex phenomenon involving a variety of dynamically regulated molecular and cellular processes whose nature is still largely unknown. Understanding the role of different proteins in controlling synapses formation and stabilization may help elucidating, at the network level, the mechanisms by which inhibitory transmission controls network excitability and oscillatory behavior, crucial for information processing in the brain.

Aim of this e-book is to highlight recent advances in these processes, bringing together leading experts in the field, who have made major contributions to our understanding of the cellular and molecular mechanisms regulating the appropriate assembly, location, and function of pre and postsynaptic specializations at inhibitory synapses.

This e-book comprises nine reviews, one perspective and three research articles organized in a logic way following the information flow from the pre to the postsynaptic site.

In the first article, Jovanovic and Thomson (2011) (School of Pharmacy, UCL) review the developmental processes determining the tangential migration of GABAergic interneurons from the ganglionic eminence to the neocortex where the formation of appropriate synapses seems to be facilitated by cell–cell recognition, most probably *via* protein–protein interactions across the synaptic cleft.

Grantyn et al. (2011) (Institute of Neurophysiology Charité, Berlin) discuss how, during postnatal development, GABAergic synapses are characterized by a high release probability, often multivesicular (ballistic) and asynchronous. The ballistic mode of operation of immature synaptic terminals might be instrumental for recruiting and stabilizing receptors *via* postsynaptic calcium signals, triggered by the depolarizing action of GABA and activation of voltage-dependent calcium channels.

Early in postnatal life, the depolarizing action of GABA is crucial for the construction of neuronal circuits. This process depends on intracellular chloride homeostasis which is under control of the cation-chloride co-transporter KCC2. In addition, by interacting with actin cytoskeleton, KCC2 exerts, independently of its role on chloride homeostasis, a crucial role on spines morphogenesis. Chamma et al. (2012) (INSERM UMR-839, Paris), summarize the functional impact that structurally different forms of KCC2 have on inhibitory and excitatory transmission, highlighting the cellular and molecular mechanisms by which neuronal activity regulates its action *via* transcriptional and post-translational modifications.

In the following research paper, Bhumbra et al. (2012) (Department of Neuroscience, Physiology and Pharmacology, UCL), convincingly demonstrate that, in contrast to neonates, in the spinal cord of juvenile animals inhibitory postsynaptic currents are entirely glycinergic, raising the possibility that the co-release of GABA and glycine is developmentally regulated.

Once released from presynaptic terminals, GABA diffuses in the synaptic cleft and binds to postsynaptic receptors. This process is very fast and occurs in non-equilibrium conditions. Barberis et al. (2011) (Department of Neuroscience, IIT and Department of Biophysics, Wroclaw University) discuss how GABA transient in the cleft influences the shape of synaptic currents. The authors describe the methods used to estimate the neurotransmitter transient using tools enabling to indirectly infer with its time course, including low affinity competitive GABA_A_ receptor antagonists or gating modifiers. GABA transient may limit the activation of postsynaptic receptors and their binding reaction to mono-ligand state, promoting low probability channel opening and fast deactivation kinetics.

The intriguing hypothesis that GABA *transporters* may exert a homeostatic control on GABA release is put forward in a perspective article by Conti et al. (2011) (Department of Neuroscience, Università Politecnica delle Marche). On the basis of the literature and recent findings the authors suggest that, in physiological conditions, vesicle filling is dominated by the GABA synthesizing enzyme GAD and by the GABA transporter GAT-1. This may be relevant for activity-dependent regulation of synaptic strength.

In the following review, Mortensen et al. (2012) (Department of Neuroscience, Physiology and Pharmacology, UCL), compare the potency measured under the same experimental conditions, of a series of GABA_A_ receptor isoforms, physiologically relevant to phasic and tonic inhibition. The highest potency would be compatible with extrasynaptic receptors, containing the α 6 subunit and exposed, during spillover of GABA from synapses, to low agonist concentrations, while the lowest potency would be compatible with synaptic GABA_A_ receptors containing the α2/α3βγ subunits, exposed, during vesicular release, to high agonist concentrations.

Using a genetic approach, Janssen et al. (2011) (Department of Pharmacology and Physiology, Georgetown University) found that the conditional deletion of β 3 subunits in D2-positive striato-pallidal medium spiny neurons led to a reduced network excitability due to a decrease in tonic GABA_A_-mediated conductance. They suggest that the β 3 subunit may be an important pharmacological target for the treatment of striatal disorders.

Next, Shrivastava et al. (2011) (Department of Biochemistry and Molecular Biology, Vienna University and Institut de Biologie, Ecole Normale Superieure, Paris), provide an overview on the mechanisms regulating the cross talk between GABA_A_ receptors and other ligand-gated ionic channels or G-protein coupled receptors co-localized on the postsynaptic membrane. The interaction may occur *via* a direct coupling between two receptors or *via* the activation of intracellular signaling pathways. Usually the interaction results in a reduced GABAergic inhibition with consequent disinhibition. A similar receptor–receptor interaction may occur also at extrasynaptic sites to regulate tonic GABA_A_-mediated inhibition.

Sassoè-Pognetto et al. (2011) (Department of Anatomy, Pharmacology and Forensic Medicine, University Torino), provide an overview of the high degree of molecular diversity of GABAergic synapses which contrasts with the apparent simplicity of their ultra-structural organization. The authors propose to develop “cataloguing tools” that should help uncovering how different proteins impact on the functional properties of the synapses and how synaptic organization changes during development.

The complex and still “enigmatic” role of the scaffolding molecule gephyrin at GABAergic synapses is exquisitely reviewed by Tretter et al. (2012) (Department of Biochemistry and Molecular Biology, Vienna University and School of Medicine, Tufts University, Boston). Gephyrin is dissected as a structural molecule responsible for trapping and concentrating GABA_A_ receptors in front of presynaptic releasing sites, by directly interacting with several types of α-containing subunits. Its heterogeneity, obtained through alternative splicing and post-translational modifications, associated with complex biophysical properties of its domains leads to the construction of diverse structural scaffolds that can accommodate different GABA_A_ receptor subtypes. Gephyrin is also recognized as a crucial hub for multiple signal transduction pathways and trans-synaptic signaling that ultimately impact on synaptic dynamics and synaptic plasticity.

Papadopoulos and Soykan (2011) (Max-Plank Institute of Experimental Medicine, Göttingen), summarize recent advances in the role played by the GDP/GTP-exchange factor collybistin in selective gephyrin transport, clustering, and maintenance at GABAergic synapses. One of the most interesting issues raised by the authors regard a comprehensive description of the putative circuit-specific role played by collybistin isoforms in selected region of the mammalian forebrain.

Finally, Jin et al. (2012) (Department of Neuroscience, Uppsala University), report changes in GABA_A_ receptors subunits mRNA in the hippocampus and cortex of *post mortem* brains of individual suffering from alcohol dependence. Interestingly, the observed changes refer mainly to extrasynaptic GABA_A_ receptors, which mediate tonic inhibition responsible for regulating basic neuronal excitability.
